# Alpha3/alpha2 power ratios relate to performance on a virtual reality shopping task in ageing adults

**DOI:** 10.3389/fnagi.2022.876832

**Published:** 2022-09-23

**Authors:** Joel Patchitt, Lilla A. Porffy, Gabriella Whomersley, Timea Szentgyorgyi, Jack Brett, Elias Mouchlianitis, Mitul A. Mehta, Judith F. Nottage, Sukhi S. Shergill

**Affiliations:** ^1^Institute of Psychiatry, Psychology & Neuroscience, King’s College London, London, United Kingdom; ^2^Trafford Centre for Medical Research, University of Sussex, Brighton, United Kingdom; ^3^Faculty of Media and Communications, Bournemouth University, Poole, United Kingdom; ^4^School of Psychology, University of East London, London, United Kingdom; ^5^Department of Psychological Sciences, Birkbeck, University of London, London, United Kingdom; ^6^Kent and Medway Medical School, Canterbury, United Kingdom; ^7^Kent and Medway National Health Service and Social Care Partnership Trust, Kent, United Kingdom

**Keywords:** virtual reality, cognition, neuropsychological testing, ageing, age-related cognitive decline, electroencephalography, alpha rhythm, cognitive markers

## Abstract

**Background:**

Aspects of cognitive function decline with age. This phenomenon is referred to as age-related cognitive decline (ARCD). Improving the understanding of these changes that occur as part of the ageing process can serve to enhance the detection of the more incapacitating neurodegenerative disorders such as Alzheimer’s disease (AD). In this study, we employ novel methods to assess ARCD by exploring the utility of the alpha3/alpha2 electroencephalogram (EEG) power ratio – a marker of AD, and a novel virtual reality (VR) functional cognition task – VStore, in discriminating between young and ageing healthy adults.

**Materials and methods:**

Twenty young individuals aged 20–30, and 20 older adults aged 60–70 took part in the study. Participants underwent resting-state EEG and completed VStore and the Cogstate Computerised Cognitive Battery. The difference in alpha3/alpha2 power ratios between the age groups was tested using *t*-test. In addition, the discriminatory accuracy of VStore and Cogstate were compared using logistic regression and overlying receiver operating characteristic (ROC) curves. Youden’s J statistic was used to establish the optimal threshold for sensitivity and specificity and model performance was evaluated with the DeLong’s test. Finally, alpha3/alpha2 power ratios were correlated with VStote and Cogstate performance.

**Results:**

The difference in alpha3/alpha2 power ratios between age cohorts was not statistically significant. On the other hand, VStore discriminated between age groups with high sensitivity (94%) and specificity (95%) The Cogstate Pre-clinical Alzheimer’s Battery achieved a sensitivity of 89% and specificity of 60%, and Cogstate Composite Score achieved a sensitivity of 83% and specificity of 85%. The differences between the discriminatory accuracy of VStore and Cogstate models were statistically significant. Finally, high alpha3/alpha2 power ratios correlated strongly with VStore (*r* = 0.73), the Cogstate Pre-clinical Alzheimer’s Battery (*r* = -0.67), and Cogstate Composite Score (*r* = -0.76).

**Conclusion:**

While we did not find evidence that the alpha3/alpha2 power ratio is elevated in healthy ageing individuals compared to young individuals, we demonstrated that VStore can classify age cohorts with high accuracy, supporting its utility in the assessment of ARCD. In addition, we found preliminary evidence that elevated alpha3/alpha2 power ratio may be linked to lower cognitive performance.

## Introduction

Aspects of cognitive function decline with age (Murman, 2015), and these changes are not uniform across cognitive domains (Glisky, 2007). Crystallised intelligence (i.e., cumulative skills and acquired knowledge) improves with age and remains largely intact until late adulthood, whilst fluid intelligence (i.e., reasoning and problem solving) gradually diminishes (Murman, 2015). Evidence suggests that some fluid abilities (e.g., reasoning, spatial visualisation) begin to decline when healthy adults reach their late twenties and early thirties (Salthouse, 2009). This phenomenon is commonly referred to as age-related cognitive decline (ARCD). Improving the understanding of these cognitive changes that occur as part of the normal ageing process can serve to enhance the detection of the more incapacitating neurodegenerative conditions such as Alzheimer’s disease (AD).

Despite increased knowledge of the brain mechanisms underlying neurodegenerative disorders, they are still primarily diagnosed using a combination of standardised neuropsychological assessments and subjective reports of cognitive decline (Stothart et al., 2021). This is because the assessment of biological markers, such as blood-based and image-based markers of beta-amyloid, tau, microglial activation, or glucose hypometabolism, often require more invasive procedures, and are high in cost and limited in availability (Frisoni et al., 2017). One technique, electroencephalography (EEG) provides a non-invasive and cost-effective approach to studying potential biomarkers. Indeed, a decline in cognition can be detected by rhythmic changes in frequency band power at rest (Babiloni et al., 2006). One promising EEG marker is the elevated relative power ratio of the high and lower alpha (α3/α2) band (Moretti et al., 2007). Increased α3/α2 power ratio was found in those patients with mild cognitive impairment (MCI) who converted to AD, but not in those who converted to non-AD dementias or did not convert during a 3-year retrospective follow-up study (Moretti et al., 2011a). In addition, increased α3/α2 power has been linked to hippocampal atrophy in patients with AD (Moretti et al., 2011b,^2012^), and cognitive decline in patients with MCI (Moretti et al., 2013). The α3/α2 power ratio, however, has never been studied in a healthy ageing population; thus, it is unclear whether it is present in ARCD or specific to MCI and AD.

Existing neuropsychological measures also have several shortcomings. Standard assessments are prone to cultural bias and test-induced anxiety (Dorenkamp and Vik, 2018; Ng et al., 2018), lack ecological validity (Chaytor and Schmitter-Edgecombe, 2003), and insensitive to early stage cognitive dysfunction (Stothart et al., 2021). The most widely used screening tool for dementia, the Mini Mental State Examination (MMSE) (Folstein et al., 1975), is susceptible to both ceiling and floor effects; its outcome is influenced by education level, language, and cultural factors; and has no utility in the assessment of complex cognitive functions (Woodford and George, 2007). Newer computerised neuropsychological assessments may be more sensitive to early stage cognitive decline. The Cogstate Pre-clinical Alzheimer’s Battery – measuring attention, processing speed, visual learning, and working memory – can reliably differentiate between healthy, MCI, and AD populations; with greater decline observed in those who carry the apolipoprotein E gene (APOE) ε4 allele, conferring increased for AD (Lim et al., 2012, 2015). However, the Cogstate Battery also has its limitations, with a recent prospective follow-up study showing that a single Cogstate assessment was not useful in predicting elevated beta-amyloid and tau levels in cognitively intact ageing individuals, while only achieving moderate diagnostic accuracy in predicting conversion from normal cognition to MCI with 77% sensitivity, 61% specificity (Stricker et al., 2020).

Cognitive assessments embedded in virtual reality (VR) may provide a solution to some of limitations associated with traditional neuropsychological testing. VR technology allows for the development of three-dimensional (3D), interactive environments that resemble real-life situations, enhancing ecological validity and engagement (Bohil et al., 2011; Parsons, 2015). 3D spatial navigation is inherent to VR (Slater and Sanchez-Vives, 2016), placing additional cognitive load on the player similar to those elicited by real-life scenarios (Armougum et al., 2019). Indeed, a meta-analytic review found that VR tasks engage a greater range of cognitive domains compared to standard measures due to their increased complexity (Negut et al., 2016a). These attributes make VR-based tests sensitive to the early detection of dysfunctions in Instrumental Activities of Daily Living associated with cognitive decline (Romero-Ayuso et al., 2021). VR tasks have also demonstrated increased sensitivity in ageing healthy adults (Negut et al., 2016b). For example, age was the strongest predictor of performance on a recently developed VR test, ECO-VR, as opposed to education level or vocabulary skills (Oliveira et al., 2018). We recently reported that a novel VR shopping task, VStore, engages cognitive domains implicated in ARCD and AD, and is highly sensitive to predicting chronological age, both as a continuous and dichotomous outcome in healthy adults (Porffy et al., 2022a).

In this study, we employ novel methods to assess cognition in young and ageing cohorts of healthy individuals. First, we assess the utility of the α3/α2 power ratio EEG marker in ARCD. We expect that ageing participants have a higher α3/α2 ratio compared to young volunteers. Second, we aim to confirm our previous findings by testing whether VStore can discriminate between the two age cohorts and compare its discriminatory accuracy to Cogstate. Finally, to establish the relationship between these functional behavioural measures and EEG marker of cognition, we assess whether participants with a higher α3/α2 ratio have a lower performance on cognitive measures assessed by VStore and Cogstate.

## Materials and methods

### Participants

A total of 40 healthy volunteers aged 20–30 (*n* = 20) and 60–70 (*n* = 20) completed the study. Participants were recruited from South London *via* advertisements in local businesses, and on social media and local community websites. Participants were excluded if they had (1) a diagnosis of any Axis I disorder (DSM-5) (American Psychiatric Association, 2013), (2) alcohol and/or substance use disorder, (3) clinically significant motion sickness, (4) a neurological illness, (5) mobility issues, or (6) were pregnant. The sample size was determined based on the difference in overall VStore performance in our previous study (Porffy et al., 2022a) – see Supplementary Material 1. Hence, we decided to recruit 20 participant per age group. This sample size would give a sensitivity to detect an effect size of 0.91 or greater in α3/α2 ratio.

### Behavioural measures

#### VStore

VStore is an immersive VR (IVR) assessment measuring functional cognition. The task takes approximately 30 min to complete including orientation (10 min), practice (10 min), and testing (10 min). Practice takes place in a virtual courtyard, where participants learn how to move around and manipulate objects. Once confident in the VR space, participants are teleported to a virtual minimarket where they complete the assessment.

**FIGURE 1:**
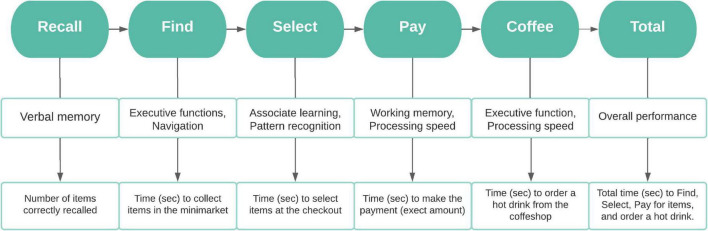
Flowchart depicting the steps required to complete VStore, its corresponding cognitive domains, and outcome variables. ©Porffy et al. (2022b). Originally published in the Journal of Medical Internet Research (https://www.jmir.org), January 26, 2022. This is an open-access article distributed under the terms of the Creative Commons Attribution License (CC BY 4.0).

At the start, 12 items are read out from a shopping list by a virtual avatar standing by the shop entrance (Table 1). The first task is to memorise and recall as many items from this list as possible. Following verbal recall, participants are presented with the shopping list and instructed to move around the minimarket and collect the items as fast and accurate as possible. Once all the items are collected, they are required to select and pay for them at a self-checkout machine, providing the exact amount. The task is completed by ordering a hot beverage from the coffee shop situated at the back of the store. The flowchart below summarises the steps required to complete VStore, its corresponding domains of cognition and outcome variables (Figure 1). Detailed information on VStore including development, feasibility, acceptability, tolerability, apparatus and software description, movement specification, and visual illustration has been published elsewhere (Porffy et al., 2022a,b).

**TABLE 1:** VStore shopping list.

1.	Cornflakes	7.	Colgate yoothpaste
2.	Tropicana orange juice	8.	Red apple
3.	Coca cola	9.	Raspberry jam
4.	Full fat milk	10.	Baked neans
5.	Tuna sandwich	11.	Orange
6.	Head and shoulders	12.	Brown bread

#### Cogstate

Cogstate is a computerised cognitive battery designed to assess cognition across multiple domains. Cogstate is simple to use; therefore, it is deemed to be suitable for assessing older adults (Zygouris and Tsolaki, 2015). For the purposes of this study, eight tasks were selected measuring processing speed, attention, working memory, visual and verbal learning, executive functions, and paired associated learning (Table 2). Four of these – Detection, Identification, One Card Learning, One-back – have been validated as a Pre-clinical Alzheimer’s Battery (Lim et al., 2012). We, therefore, used these tasks to calculate the Pre-clinical Alzheimer’s Battery score by standardising individual task scores and then averaging them within the whole sample including both young and ageing cohorts. We also generated a Composite Cogstate Score with the same procedure including all eight tasks.

**TABLE 2:** List of cogstate tasks, corresponding cognitive domains, and main outcome measures.

**Code**	**Task name**	**Cognitive domain**	**Outcome (metric)**
DET	Detection	Processing speed	Reaction time (log 10 ms)
IDN	Identification	Attention	Reaction time (log 10 ms)
OCL	One Card Learning	Visual learning	Accuracy (arcsine proportion)
ONB	One-Back	Working memory	Reaction time (log 10 ms)
TWO	Two-Back	Working memory	Accuracy (arcsine proportion)
CPAL	Continuous Paired Associate Learning	Paired associate learning	Total number of errors
GMLT	Groton Maze Learning Task	Executive functions	Total number of errors
ISLT	International Shopping List	Verbal learning	Number of correct responses

#### Wechsler abbreviated scale of intelligence I

Intelligence quotient (IQ) was measured by the abbreviated version of the Wechsler Adult Intelligence Scale – first edition (Wechsler, 1999). The Wechsler Abbreviated Scale of Intelligence I (WASI-I) combines crystallised and fluid abilities to derive an age relative IQ score. The subtests used included the matrix reasoning and vocabulary tests.

#### Technological familiarity questionnaire

We developed a short questionnaire assessing technological familiarity (Porffy et al., 2022a,b). The Technological Familiarity Questionnaire (TFQ) includes 13 questions ascertaining the frequency, comfort, and ability in technology use on a 5-point scale. Higher scores indicate greater technological familiarity. In the present study, the TFQ total score was used to index of overall technological ability, while the item “how often do you use VR” was used to compare the frequency of VR use between age cohorts. The questionnaire’s internal consistency was adequate (α = 0.83, 95% CI = 0.82–0.89).

### EEG resting-state recording

EEG recordings took place between 10:00–12:00 to control for time dependent electromagnetic fluctuations (Croce et al., 2018). Data was acquired using Compumedics Neuroscan SynAmps 64-channel amplifier. Participants were seated in a comfortable chair located in an unlit Faraday Cage with a computer monitor – displaying a fixation cross – 75 cm from their eyes. EEG measurements were taken using a 64 electrode Easycap, grounded to the AFz electrode. The channels were arranged according to the standard 10–10 system, except that some of the central electrodes (FC1, FC2, C1, C2, CP1, and CP2) were replaced by sub-temporal electrodes (FT9, FT10, P9, P10, POO9, and POO10) to better capture inferior temporal signals. All electrodes were referenced to the left mastoid. Vertical and horizontal eye movements were accounted for using an electrode above and below the right eye, and one on the outer canthus of the left eye. Impedance levels were reduced below 5 kΩ. Total recording time reached 4 min 30 s consisting of eyes open and closed data. On average, 2 min 30 s of eyes closed data were obtained per recording. Participants were instructed through a headphone to open or close their eyes in 30 s intervals to reduce fatigue and the slowing of alpha waves (Ranlund et al., 2014). Data were acquired using a band pass filter of 0–1,000 Hz at a sampling rate of 5,000 Hz, and later digitised using a band-pass filter of 0.3–70 Hz at a sampling rate of 250 Hz as per previous studies in this frequency range (Moretti et al., 2012).

### Procedures

Potential participants were pre-screened over the phone. Those eligible were invited for a single, 4-hour-long study visit. Informed consent was obtained at the start, followed by demographics and a brief mental and physical health history. EEG recordings were taken first, and the TFQ was completed during cap fitting. Upon acquisition of the EEG recording, participants were given a break to wash their hair and take lunch. Following the break, VStore and Cogstate were administered in a counterbalanced fashion, to minimise order effect. Finally, participants completed the WASI. Volunteers were compensated for their time and reimbursed for their travel and sustenance. Ethical approval was granted by the Psychiatry, Nursing and Midwifery Research Ethics Committee, King’s College London (HR-18/19-11868).

### Analysis

#### Behavioural data

Prior to data analysis, VStore outcomes measured in seconds were log transformed to stabilise variance. Outliers for all main VStore and Cogstate outcomes were defined using 2.5 standard deviations (SDs) above or below the sample mean as cut-off. Two participants, both in the 60–70 group, with outlier values on more than one VStore outcome were completely removed from all analyses. Additionally, a single extreme outlier value was removed from the Two-Back Cogstate task (*x* = 0.51, normal range = 0.84–1.77). For that participant, the Cogstate Composite Score was calculated based on average performance across the remaining 7 tasks. Furthermore, we found 3 more outliers among main outcome variables – one on VStore Pay, one on Cogstate Detection, and one on Cogstate Groton Maze Learning Task (Supplementary Material 2). For these, sensitivity analysis was carried out to establish whether they had impacted results. Descriptive statistics for VStore and Cogstate are presented in the supplementary (Supplementary Material 3–5).

Differences in demographic characteristics between age cohorts were tested using independent samples *t*-test and Chi-square. Group differences in cognitive performance – as measured by the Pre-Clinical Alzheimer’s Battery, Cogstate Composite Score, and main VStore outcomes – were tested using independent samples *t*-test. The alpha level was adjusted using Bonferroni correction. Differences in VStore outcomes were considered significant at α = 0.009, and at α = 0.025 for Cogstate. Bootstrapped effect sizes (nboot = 1000) were calculated for each outcome using Hedges’s *g*. To establish VStore’s discriminatory accuracy, we built a logistic regression model using VStore Total Time as a predictor of group status (GroupStatus ∼ VStore_T_Total). VStore Total Time was selected to represent a composite measure of overall performance. We repeated the procedure for the Cogstate batteries (GroupStatus ∼ Cogstate_Alz; GroupStatus ∼ Cogstate_Comp) and generated three overlying ROC curves. The analysis was repeated with the TFQ included to test its influence on results. Youden’s J statistic was used to establish the optimal threshold for sensitivity and specificity and model performance was compared with the DeLong’s test.

#### EEG data

Pre-processing and spectral analysis were carried out in EEGLAB and MATLAB (Delorme and Makeig, 2004; MATLAB, 2021). We removed ocular and power-line noise using independent component analysis. Next, we re-referenced to the average of left and right mastoids, and manually rejected any remaining artefacts and eyes open data. A Fast Fourier Transform-based power spectrum analysis calculated the spectral power density of the EEG rhythms with a frequency range of 2–40 Hz and a 0.5 Hz frequency resolution. The theta/alpha (θ/α) transition frequency (TF) and individual α frequency (IAF) were used as anchors to identify the subdivisions of the extended alpha spectrum (Klimesch, 1999). TF was computed as the minimum power within the extended α range (5–14 Hz) across all 64 channels. This identified where the θ/α frequencies intersect. IAF was computed as the highest averaged power, representing the “peak” within the same α frequency range. Using the TF and IAF, we isolated the absolute α3 and α2 band subdivisions. This was done by averaging the power values between the IAF to IAF + 2 hz for the α3 frequency band and calculating the same average between the middle point of the TF-IAF range to the IAF peak for the α2 frequency band (Moretti et al., 2007). Finally, relative spectral power was calculated for both for α3 and α2 band subdivisions by first, using the ratio between the absolute power for each frequency bin and mean spectra power from 2–45 hz, and then establishing the mean relative band powers from each frequency bin in that specific frequency band. The EEG dataset consisted of 35 participants. One participant was an outlier on both VStore and α3/α2 spectral power ratio outcomes (i.e., negative value for α3/α2), one person did not complete the EEG recording, and further three participants were excluded based on poor EEG data quality (i.e., no clear IAF peak). In total, 4 participants were removed from the 60–70 cohort and 1 participant was removed from the 20–30 cohort.

The remaining sample was assessed further for outliers. Values for two participants were found to be 2.5 SDs above the group mean (Supplementary Material 2). Again, sensitivity analysis was applied to establish their influence on results. First, α3/α2 power ratios were compared between age cohorts using independent samples *t*-test. The effect size was calculated using Hedges’ g. Second, Pearson’s correlations were used to assess the associations between the α3/α2 ratios and VStore Total Time, and the Cogstate Pre-clinical Alzheimer’s Battery and Composite Scores across the total sample. As per previous research (Moretti et al., 2012, 2013; Moretti, 2015b), we then grouped α3/α2 power ratios according to increasing tertiles. The boundaries were selected to create three equal groups classified as low (α3/α2 < 0.94, *n* = 12), mid (0.94 ≤ α3/α2 ≤ 1.02, *n* = 11), and high (α3/α2 ≥ 1.02, *n* = 12) α ratio. Chi-square test was used to assess the relationship between α3/α2 tertile groups and age cohort status. Finally, grouped α3/α2 ratios were separately correlated with VStote Total Time and the Cogstate batteries to establish how they relate to cognitive performance.

## Results

### Demographics

Demographic information for both cohorts is presented in Table 3. There was a significant difference in IQ and technological familiarity between groups. However, the frequency of past VR use was not higher in the 20–30 age cohort (16 never used VR) compared to the 60–70 age cohort (15 never used VR), *X*^2^_(1)_ = 0, *p* = 1. The 60–70 age group only included people from a White ethnic background (*n* = 18, 100%), the 20–30 age group also included individuals from Asian (*n* = 5, 25%) and Mixed (*n* = 3, 15%) backgrounds.

**TABLE 3:** Sample characteristics.

	20–30	60–70	Statistics
*N*, count (%)	20 (53%)	18 (47%)	–
Age, mean (SD)	24.1 (3.0)	65.8 (2.4)	–
Gender, count (%)	10F (50%)	9F (50%)	*X*^2^_(1)_ = 0, *p* = 1
IQ, mean (SD)	117.6 (8.2)	125.3 (7.1)	*t*_(35_._9)_ = –3.085, *p* = 0.004
Education years, mean (SD)	16.1 (2.5)	16.2 (3.5)	*t*_(30_._1)_ = –0.172, *p* = 0.865
Technological familiarity, mean (SD)	45.9 (6.2)	39.1 (9.0)	*t*_(29_._8)_ = 2.570, *p* = 0.012

### Group differences in cognitive performance

The 60–70 age cohort were significantly slower on VStore outcomes Find, Select, Coffee, and Total Time compared to the 20–30 age cohort (Table 4). In addition, older volunteers achieved a lower score on the Cogstate Pre-clinical Alzheimer’s Battery and had a lower Cogstate Composite Score. Results were unchanged following the removal of outliers. To further assess how performance on VStore relates to age in the 60–70 cohort, we run a Person’s correlation between age and VStore Total Time (Supplementary Material 6).

**TABLE 4:** Group differences in cognitive performance between participants aged 20–30 vs. 60–70.

	20–30 Mean	60–70 Mean	*t*	*p*-value	Hedges’ *g* (95% CI)
VStore recall	6.25	5.50	1.103	0.278	0.35 (-0.40–0.99)
VStore find	5.84	6.27	7.189	< 0.001	2.29 (1.38–3.03)
VStore select	4.63	5.10	4.974	< 0.001	1.58 (0.55–2.38)
VStore pay	2.90	3.07	1.280	0.209	0.41 (-1.05–0.38)
VStore coffee	3.34	3.78	3.902	< 0.001	1.23 (0.40–1.93)
VStore total	6.21	6.64	7.841	< 0.001	2.48 (1.50–3.25)
Cogstate alzheimer	0.42	–0.41	3.689	< 0.001	1.19 (0.50–1.80)
Cogstate composite	0.47	–0.45	4.496	< 0.001	1.44 (0.54–2.17)

Recall is presented as the number of correct responses. Find, Select, Pay, Coffee, and Total are presented in log transformed seconds.

### Group differences in α3/α2 ratio

The α3/α2 power ratios in the 20–30 age cohort (mean = 0.97, SD = 0.09) did not significantly differ from α3/α2 power ratios in the 60–70 cohort (mean = 1.07, SD = 0.26), *t*_(18_._3)_ = -1.514, *p* = 0.147 (Hedges’ *g* = -0.518, 95% = -1.14–0.12).

### Age cohort classification

**FIGURE 2:**
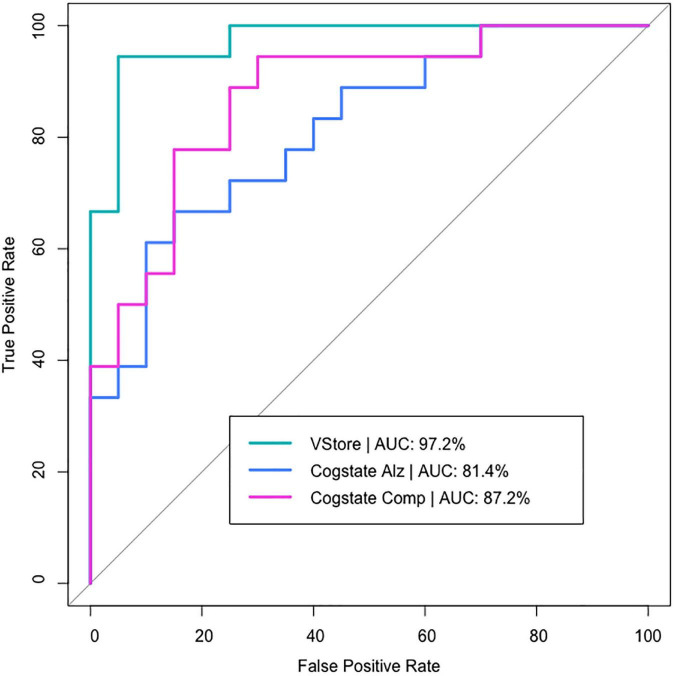
VStore and Cogstate models predicting age group belonging.

Figure 2 presents the discriminatory accuracy for VStore and Cogstate. VStore achieved a sensitivity of 94% and specificity of 95% at the optimal threshold of 0.47. The Cogstate Pre-clinical Alzheimer’s Battery achieved a sensitivity of 89% and specificity of 60% at the optimal threshold of 0.37. Finally, the Cogstate Composite Score achieved a sensitivity of 83% and specificity of 85% at the optimal threshold of 0.38. The differences between the VStore model and both Cogstate models were statistically significant (Pre-Clinical Alzheimer’s Battery: *z* = 2.498, *p*-value = 0.013; Cogstate Composite Score: *z* = 2.020, *p*-value = 0.043). Outliers did not have an impact on results, and the inclusion of the TFQ did not alter VStore findings. However, the TFQ decreased sensitivity (67%) and increased specificity (85%) for the Cogstate Pre-clinical Alzheimer’s Battery, and increased sensitivity (94%) and decreased specificity (70%) for the Cogstate Composite Score. The AUC remained similar for all 3 models (< 1% difference).

### Relationship between α3/α2 power ratios and cognitive performance

**FIGURE 3:**
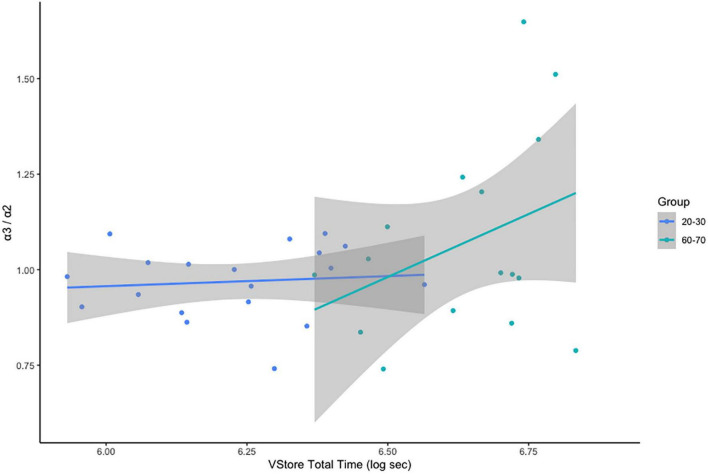
Correlations between VStore Total Time and α3/α2 ratios stratified by age group.

Across the complete sample, a significant positive association was found between α3/α2 power ratios and VStore Total Time; r = 0.35, *p* = 0.04. This finding was primarily driven by the moderate association between VStore Total Time and α3/α2 power ratios in the 60–70 cohort: *r* = 0.36, *p* = 0.17 (Figure 3). The relationship between VStore Total Time and α3/α2 power ratios; however, was no longer significant following the removal of outlier values; *r* = 0.16, *p* = 0.38. No correlation was found between α3/α2 power ratios and the Cogstate Composite Score; *r* = -0.14, *p* = 0.43; or Cogstate Pre-clinical Alzheimer’s Battery; *r* = -0.13, *p* = 0.45.

**FIGURE 4:**
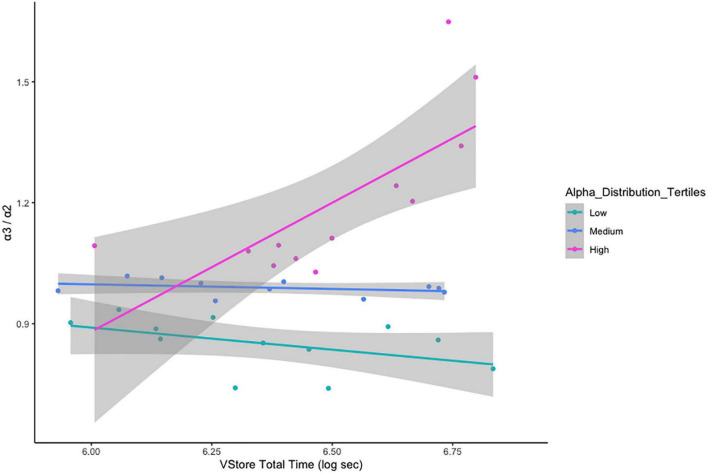
Correlations between VStore Total Time and α3/α2 ratios stratified by tertiles.

**FIGURE 5:**
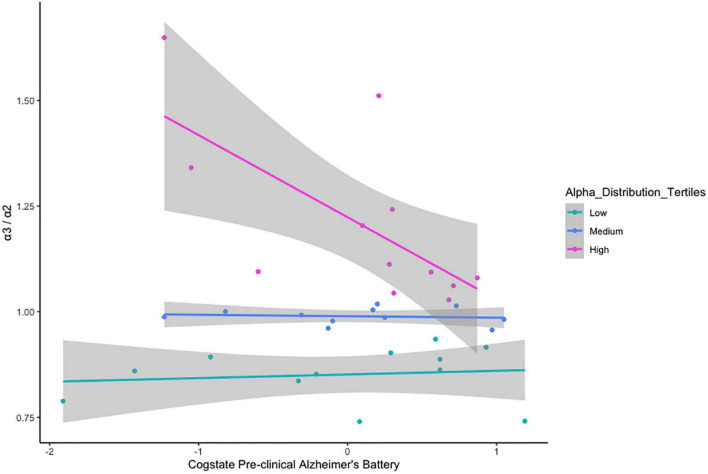
Correlations between the Pre-clinical Alzheimer’s Battery and α3/α2 ratios stratified by tertiles.

**FIGURE 6:**
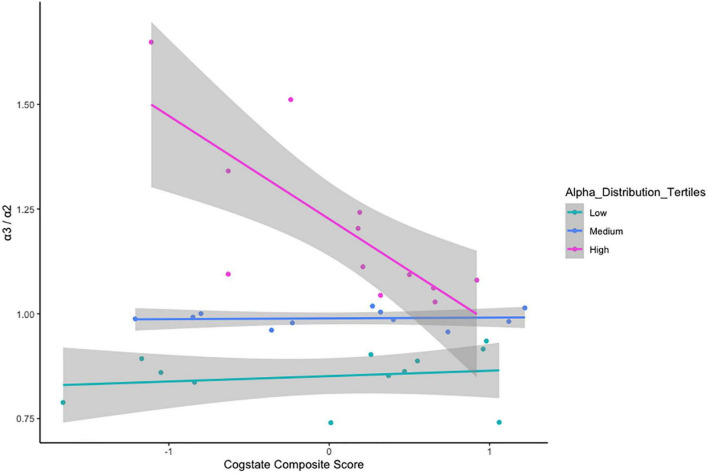
Correlations between the Cogstate Composite Score and α3/α2 ratios stratified by tertiles.

There was no relationship between α3/α2 ratio classification and age cohort status, *x*^2^_(2)_ = 1.237, *p* = 0.538 (Table 5). VStore Total Time showed a significant positive correlation with α3/α2 power ratios classified as high (*r* = 0.73, *p* = 0.01), but not with ratios in the mid (*r* = -0.32, *p* = 0.34), and low (*r* = -0.46, *p* = 0.13) ranges (Figure 4). A similar trend was observed with the Cogstate Pre-clinical Alzheimer’s Battery, showing a significant association with high α3/α2 power ratios (*r* = -0.67, *p* = 0.02), but not with ratios in the mid (*r* = -0.12, *p* = 0.72), and low (*r* = 0.13, *p* = 0.69) ranges (Figure 5). The Cogstate Composite Score also showed a significant relationship with high α3/α2 power ratios (*r* = -0.76, *p* < 0.001), but not with mid (*r* = 0.08, *p* = 0.82), and low (*r* = 0.19, *p* = 0.56) ratios (Figure 6). These results were somewhat altered following the removal of outliers, showing that VStore Total Time had the strongest association with the high α3/α2 power ratios (*r* = 0.74, *p* = 0.01), followed by the Cogstate Pre-clinical Alzheimer’s Battery (*r* = -0.64, *p* = 0.04), and Cogstate Composite Score (*r* = -0.58, *p* = 0.06).

**TABLE 5:** Frequency distributions of α3/2 power ratio tertiles by age group.

Cohorts	α3/2 tertiles			
	Low	Medium	High	Total
20–30	7	7	5	19
60–70	5	4	7	16
Total	12	11	12	35

## Discussion

To our knowledge, this is the first study to investigate α3/α2 resting state spectral power ratios in a healthy ageing cohort. Previous studies found elevated α3/α2 power ratios in individuals diagnosed with MCI and AD (Moretti et al., 2011b). We were unable to replicate these findings in healthy ageing adults. Previous, studies also showed that a high α3/α2 spectral power ratio is related to hippocampal atrophy (Moretti, 2015a). Given that participants in this study were high functioning, healthy adults, age-related changes may not have been pronounced enough to alter α3/α2 power. It is possible that the association between ageing and the high α3/α2 spectral power ratio is due to a degenerative process and is a marker of disease (Moretti et al., 2011a). Although to test this hypothesis, another sample of healthy participants and patients with MCI and AD would have to be tested on the EEG marker, hippocampal atrophy (Frisoni et al., 2008), and cognitive outcomes. Alternatively, negative findings could be due the lack of power to detect a difference in α3/α2 power ratios between age cohorts. In support, in healthy ageing individuals, evidence suggests that clusterin (CLU) gene polymorphisms, which increases AD risk, elevates α3 absolute power (Ponomareva et al., 2013). In addition, ageing has been linked to a decrease in α2 frequency power in multiple regions including limbic areas (Babiloni et al., 2006).

Receiver operating characteristic curve analysis revealed that VStore is highly sensitive and specific to the classification of the two age cohorts, more so than Cogstate, supporting its potential utility in the assessment of ARCD. These results confirm our previous findings (Porffy et al., 2022a), adding to a body of evidence that VR assessments may have an increased sensitivity in ageing healthy adults (Negut et al., 2016b). Indeed, the older cohort were slower to complete all VStore tasks, except making the payment. The VStore Pay outcome – engaging working memory, executive functions, verbal learning, and requiring fast processing speed – was amongst the weakest predictors of age as a continuous outcome in the previous study (Porffy et al., 2022a). This outcome, therefore, is likely to be less sensitive in ARCD. Interestingly, there was no marked difference between age cohorts in the number of VStore items correctly recalled. This is somewhat unexpected as verbal episodic memory tends to decline with age (Celsis, 2000). The lack of significant finding may be explained by the fact that the older cohort had a high verbal IQ on average, which may protect against memory decline (Boyle et al., 2021). Alternatively, immediate recall may not be as sensitive to cognitive decline as delayed recall (Gomar et al., 2011). Taken together, these findings suggest that assessments embedded in VR are sensitive to cognitive decline associated with ageing (Negut et al., 2016b; Oliveira et al., 2018; Porffy et al., 2022a), and also potentially provide valid concurrent measurement of everyday functioning (Romero-Ayuso et al., 2021); therefore, utilising this technology may enhance the early detection of subtle changes in cognition and related functional decline.

When we combined the EEG data from both cohorts, α3/α2 power ratios classified as high (≥ 1.02) showed a strong positive correlation with VStore Total Time, and strong negative associations with the Cogstate Pre-clinical Alzheimer’s Battery and Composite Score. This suggests that elevated α3/α2 power ratios may be related to poorer cognitive performance. The relationship between α3/α2 power ratios and cognition has not been directly investigated in healthy individuals. However, in MCI those with a high α3/α2 power ratio, cognitive test performance correlates with cortical thickness (Moretti et al., 2013), providing indirect evidence that increased α3/α2 power may relate to a decline in cognition. Generally, changes in alpha power are linked to ageing as well as neurogenerative disorders, and lower alpha frequency correlates with poor memory and slow speed of processing (Klimesch, 1999). Alpha oscillation has also been linked to cognitive test performance on tasks probing attention, episodic memory, and executive functions in subjective and MCI (Babiloni et al., 2010). These domains are engaged during VStore (Porffy et al., 2022a), which may explain the strong relationship between the EEG marker and VStore performance. Taken together, these findings suggest that VStore may be a useful screening tool for those whose α3/α2 power ratios may be elevated due hippocampal atrophy. However, this theory would have to tested.

There are some limitations to consider. VStore’s high discriminatory accuracy may partly be due lower levels of technological proficiency in ageing adults (Olson et al., 2011). Indeed, ageing participants scored lower on technological familiarity. We tried to attenuate any potential confounding effects by providing sufficient practice time prior to assessment. In addition, we reran discriminatory models with the TFQ included. The addition of the TFQ did not have an impact on VStore results, suggesting that the high discriminatory accuracy was not simply due to differences in technological familiarity. This is in line with our previous findings indicating that the variance associated with technological familiarity is already captured in statistical models of VStore (Porffy et al., 2022a). In addition, we reported that past VR use was not more frequent in the young age group relative to the ageing cohort. Nonetheless, we cannot rule out that VStore, like any other digital assessment, may potentially underestimate the cognitive abilities of older adults. Furthermore, the sample included self-selecting ageing individuals and young students and professionals; therefore, it may not be completely representative of the general population. Opportunistic sampling tends to capture volunteers who are physically and mentally capable of attending and likely to exclude low functioning agers. Such problems are pervasive in this type of research (Murman, 2015), and could have affected EEG findings.

In conclusion, we did not find evidence that the α3/α2 spectral power ratio is elevated in healthy ageing individuals compared to young individuals. However, this may be due to the lack of sufficient statistical power, or the relatively high functioning of our volunteers. Further studies comparing cognitively intact young and ageing adults to patients with MCI and AD are needed to establish whether an increase in α3/α2 spectral power ratio is present in healthy ageing, supplemented with the measurement of hippocampal volumes. Despite these limitations, we confirmed previous findings showing that VStore can classify age cohorts with high accuracy, further supporting its utility in the assessment of ARCD. High-quality prospective studies are needed to establish whether VStore and other similar IVR assessments can detect early stage cognitive decline and provide a prognostic value in predicating transition to MCI and AD. Finally, we were able to establish that an increase in α3/α2 power ratio has a strong association to lower cognitive performance, suggesting that the marker may generally be useful in assessing cognitive decline.

## Data availability statement

The datasets presented in this article are not readily available due to commercial restrictions. VStore is a propriety software. Requests to access the datasets should be directed to LP.

## Ethics statement

The study involving human participants was reviewed and approved by the Psychiatry, Nursing and Midwifery Research Ethics Committee, King’s College London (HR-18/19-11868). The participants provided their written informed consent to participate in this study.

## Author contributions

JP: study and EEG protocol design, data collection and analysis, and contributed manuscript. LP: study design, data collection and analysis, and written manuscript. GW and TS: recruitment, data collection, and read and edited the manuscript. JB: VStore software development, and read and edited manuscript. EM: manuscript preparation and review. MM: advised on data analysis, and manuscript preparation and review. JN: EEG protocol design, advised on EEG data analysis, and read and edited manuscript. SS: study design and manuscript preparation and review. All authors contributed to the article and approved the submitted version.
