# Supplementary material for: Alpha3/alpha2 power ratios relate to performance on a virtual reality shopping task in ageing adults
Source: Front Aging Neurosci. 2022 Sep 23;14:876832. doi: 10.3389/fnagi.2022.876832 (PMC9540381; doi:10.3389/fnagi.2022.876832)

**Alpha3/Alpha2 Power Ratios Relate to Performance**

**on a Virtual Reality Shopping Task in Ageing Adults**

***Supplementary Material***

**Supplementary Material 1.** Sample size calculation.

We calculated the required sample size based on the difference in VStore Total Time between participants aged 20-29 and 60-69 in our previous study (Porffy et al., 2022a); t = 5.11, p < .001, Cohen’s d = 1.67 (95%CI = 0.96-2.81). We used G*Power (version 3.1.9.3) and set the parameters as follows: tail = two, effect size (d) = 1, α = .05, 1- β = 0.8, allocation ratio = 1:1. This resulted in the minimum sample size of 34 in total – 17 participants per group.

**Supplementary Material 2.** Outliers on main outcome variables considered for sensitivity analysis.

|  | Mean | SD | Lower limit | Upper limit | Outlier value |
| --- | --- | --- | --- | --- | --- |
| DET | 1.51 | 0.11 | 2.28 | 2.82 | 2.85 |
| GMLT | 42.95 | 12.68 | 11.26 | 74.64 | 75 |
| VStore Pay | 2.98 | 0.43 | 1.91 | 4.06 | 4.09 |
| Alpha 3/2 | 1.02 | 0.19 | 0.54 | 1.50 | 1.51 |
| Alpha 3/2 | 1.02 | 0.19 | 0.54 | 1.50 | 1.65 |
| Notes. DET, Detection (Processing speed); GMLT, Groton Maze Learning (Executive function). | | | | | |

**Supplementary Material 3.** Descriptive statistics for main VStore outcomes (outliers included).

| Group | Recall  Mean (SD) | Find  Mean (SD) | Select  Mean (SD) | Pay  Mean (SD) | Coffee  Mean (SD) | Total  Mean (SD) |
| --- | --- | --- | --- | --- | --- | --- |
| 20-30 | 6.3 (2.2) | 5.84 (0.17) | 4.63 (0.30) | 2.90 (0.46) | 3.34 (0.40) | 6.21 (0.18) |
| 60-70 | 5.5 (2.0) | 6.27 (0.19) | 5.10 (0.27) | 3.07 (0.38) | 3.78 (0.29) | 6.64 (0.15) |
| Notes. Recall is presented as the number of correct responses. Find, Select, Pay, Coffee, and Total are presented in log transformed seconds. | | | | | | |

**Supplementary Material 4.** Descriptive statistics for main Cogstate outcomes (outliers included).

| Group | DET  Mean (SD) | IDN  Mean (SD) | OCL  Mean (SD) | ONB  Mean (SD) | TWO Mean (SD) | GMLT Mean (SD) | CPAL Mean (SD) | ISLT Mean (SD) |
| --- | --- | --- | --- | --- | --- | --- | --- | --- |
| 20-29 | 2.51 (0.07) | 2.67 (0.05) | 1.40 (0.08) | 2.84 (0.08) | 1.37 (0.14) | 37.75 (10.52) | 42.50 (36.55) | 29.15 (3.76) |
| 60-69 | 2.59 (0.13) | 2.74 (0.08) | 1.02 (0.09) | 2.90 (0.12) | 1.28 (0.12) | 48.72 (12.61) | 80.28 (42.77) | 25.00 (3.94) |
| Notes. DET, Detection (Processing speed); IDN, Identification (Attention); OCL, One Card Learning (Visual learning); ONB, One–back (Working memory); TWO, Two–back (Working memory); GMLT, Groton Maze Learning (Executive function); CPAL, Continuous Paired Associate Learning; ISLT, International Shopping List Task (Verbal learning). | | | | | | | | |

**Supplementary Material 5.** Descriptive statistics for the Pre-Clinical Alzheimer’s Battery and Cogstate Composite Score.

| Group | Pre-Clinical Alzheimer’s Battery  Mean (SD) | Cogstate Composite Score  Mean (SD) |
| --- | --- | --- |
| 20-30 | 0.42 (0.54) | 0.47 (0.55) |
| 60-70 | -0.41 (0.80) | -0.45 (0.69) |

**Supplementary Material 6.** Correlation between Age and VStore Total Time in the 60-70 age group.


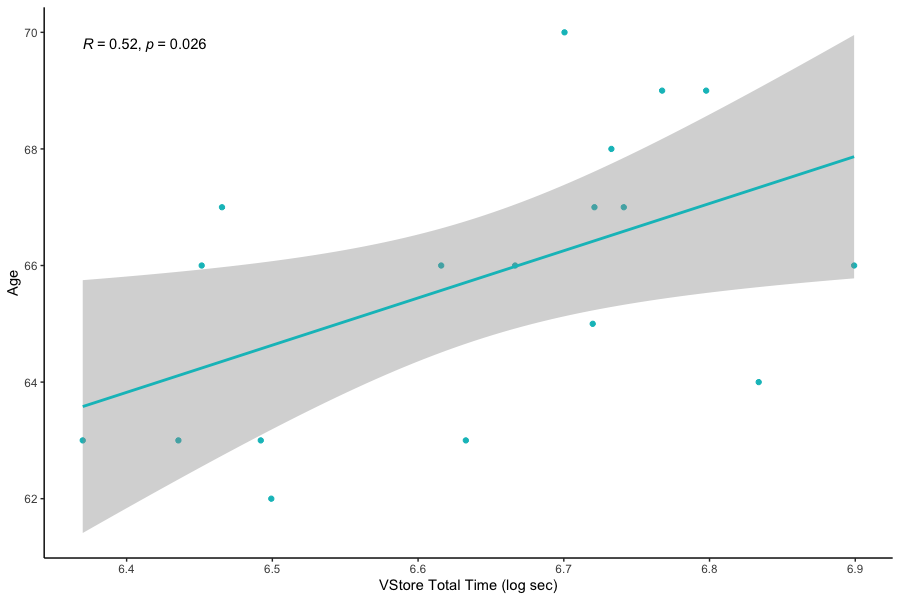

Supplement: Supplementary file 1 [file Data_Sheet_1.docx]
